# AI Methods for Antimicrobial Peptides: Progress and Challenges

**DOI:** 10.1111/1751-7915.70072

**Published:** 2025-01-04

**Authors:** Carlos A. Brizuela, Gary Liu, Jonathan M. Stokes, Cesar de la Fuente‐Nunez

**Affiliations:** ^1^ Department of Computer Science CICESE Research Center Ensenada Mexico; ^2^ Department of Biochemistry and Biomedical Sciences, Michael G. DeGroote Institute for Infectious Disease Research, David Braley Centre for Antibiotic Discovery McMaster University Hamilton Ontario Canada; ^3^ Machine Biology Group, Department of Psychiatry and Microbiology, Institute for Biomedical Informatics, Institute for Translational Medicine and Therapeutics, Perelman School of Medicine University of Pennsylvania Philadelphia Pennsylvania USA; ^4^ Department of Bioengineering and Chemical and Biomolecular Engineering, School of Engineering and Applied Science University of Pennsylvania Philadelphia Pennsylvania USA; ^5^ Department of Chemistry, School of Arts and Sciences University of Pennsylvania Philadelphia Pennsylvania USA; ^6^ Penn Institute for Computational Science University of Pennsylvania Philadelphia Pennsylvania USA

## Abstract

Antimicrobial peptides (AMPs) are promising candidates to combat multidrug‐resistant pathogens. However, the high cost of extensive wet‐lab screening has made AI methods for identifying and designing AMPs increasingly important, with machine learning (ML) techniques playing a crucial role. AI approaches have recently revolutionised this field by accelerating the discovery of new peptides with anti‐infective activity, particularly in preclinical mouse models. Initially, classical ML approaches dominated the field, but recently there has been a shift towards deep learning (DL) models. Despite significant contributions, existing reviews have not thoroughly explored the potential of large language models (LLMs), graph neural networks (GNNs) and structure‐guided AMP discovery and design. This review aims to fill that gap by providing a comprehensive overview of the latest advancements, challenges and opportunities in using AI methods, with a particular emphasis on LLMs, GNNs and structure‐guided design. We discuss the limitations of current approaches and highlight the most relevant topics to address in the coming years for AMP discovery and design.

## Graphical abstract

AI methods have become fundamental to AMP discovery and design. Classical ML approaches are now replaced or complemented by DL‐based, large protein language models, graph neural networks and structure‐guided design. This review covers recent advancements, challenges and future opportunities in AMP discovery and design using these AI techniques.

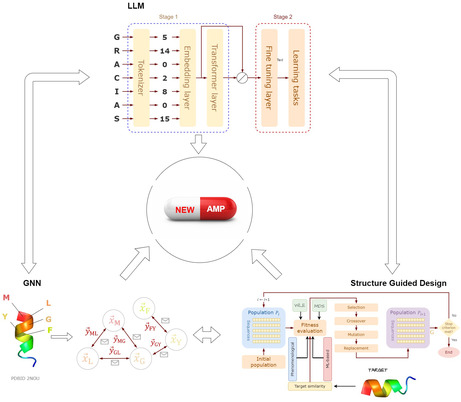

## 1 Introduction

Discovering new antibiotics, including peptides, is a time‐consuming process that can take years and incur significant costs. However, AI has recently accelerated scientific discovery, transforming this field. The discovery of novel AMPs has emerged as a promising application of machine learning. AMPs exhibit a broader spectrum of activity and reduced likelihood of resistance development compared to other antibacterial agents (Ageitos et al. 2017; Lázár et al. 2018; Nuti et al. 2017; Reddy, Yedery, and Aranha 2004). Moreover, AMPs can be engineered to enhance their therapeutic properties while minimising toxicity and cost, making them a versatile option for developing new antibiotics (Tew et al. 2010). Overall, the multifaceted benefits of AMPs, including broad‐spectrum efficacy and innovative discovery methods, make them a promising approach for combatting against antimicrobial resistance (AMR).

The history and development of AMPs are extensively reviewed by Bahar and Ren (2013), while their potential as therapeutic agents has been surveyed by Seo et al. (2012). Another recent review (Huan et al. 2020) analyses the progress in AMP research, covering their classification, mechanism of action, design methods and the influence of environmental factors such as metal ions, pH and proteases on their functions, providing a comprehensive perspective on the field.

In the broader context of infectious diseases (Wong, de la Fuente‐Nunez, and Collins 2023) discuss approaches for detecting, treating and understanding these diseases, highlighting the advances made possible by AI in each case. Specifically, in the context of antibiotics and AMPs, several key articles provide an overview of advanced design strategies (Aguilera‐Puga et al. 2024; Cesaro et al. 2023; Coelho, Santos‐Júnior, and de la Fuente‐Nunez 2024; Melo, Maasch, and de la Fuente‐Nunez 2021; Torres et al. 2021; Torres and de la Fuente‐Nunez 2019; Wan, Wong, et al. 2024). These studies collectively highlight the significant progress and innovative approaches being developed to enhance computational techniques for antibiotic discovery and other applications. Yan et al. (2022) review shallow and deep learning models for AMP discovery, although without direct comparison between the approaches. Fernandes et al. (2023) provide an overview of graph neural networks (GNNs) under the umbrella of deep geometric learning. Moreover, AMP design approaches based on deep generative models have been presented by Wan, Kontogiorgos‐Heintz, and de la Fuente‐Nunez (2022).

Despite the extensive analysis of previous and current work, several relevant aspects remain unexplored. First, a common framework that integrates similar approaches under the same umbrella is missing. Second, the potential of exploiting large language models (LLMs) in both discriminative and generative tasks, as well as the recently proposed diffusion models, has not been addressed. Third, the opportunities that GNNs present for AMP discovery and design as a general framework are also overlooked. Fourth, structure‐based design approaches have not been extensively covered.

This review seeks to provide readers with the necessary context to understand the latest advancements, challenges and opportunities in the discovery and design of AMPs using artificial intelligence models and techniques. We emphasise the roles of LLMS, GNNs and structure‐guided design. We briefly describe the challenges and opportunities related to databases, descriptors and state‐of‐the‐art machine learning models for identifying and designing AMPs.

## 2 AMP Identification and Design

**FIGURE 1:**
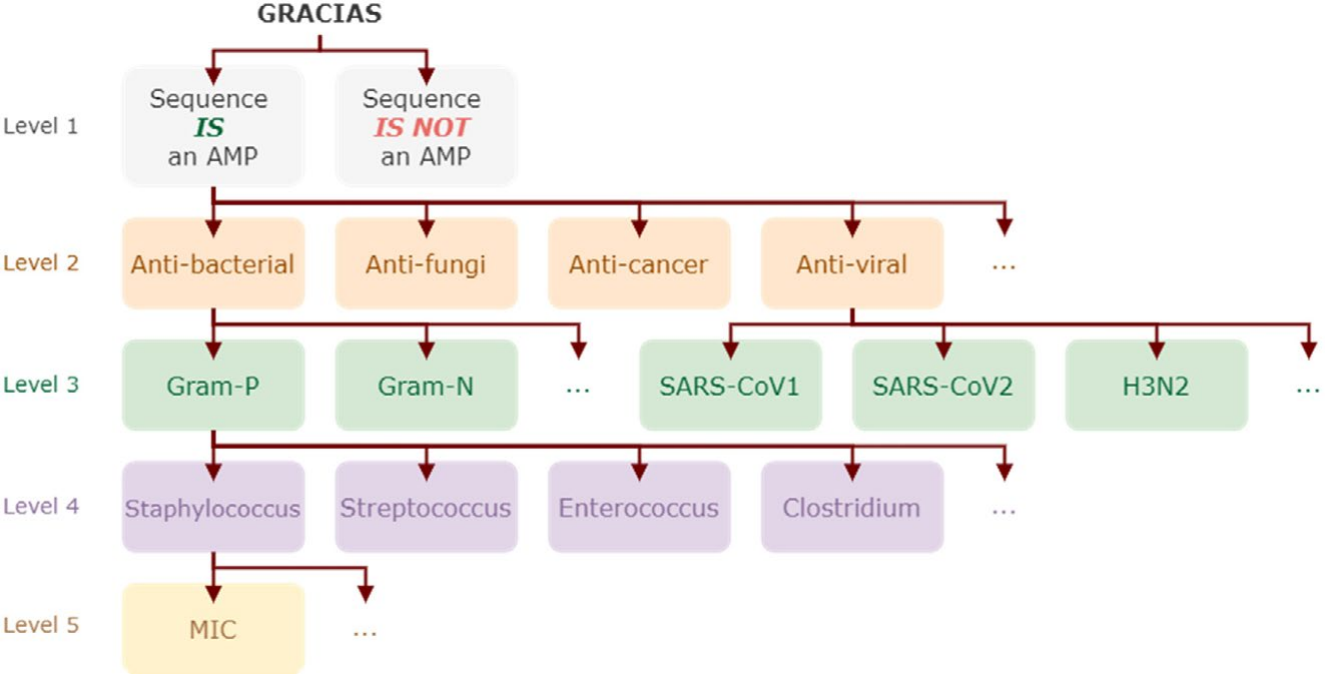
Hierarchical diagram for AMP identification. An example input sequence is ‘GRACIAS’. The first level requires discrimination between AMPs and non‐AMPs. At the second level, the target is predicted to be active against viruses, bacteria and/or fungi. The third level asks for an additional layer of granularity, such as distinguishing between Gram‐positive and Gram‐negative bacteria. The fourth level further discriminates between species. Finally, the fifth level provides detailed information on the minimum inhibitory concentration (MIC).

The ultimate goal of in silico AMP discovery is to solve the following problem of sequence‐to‐function prediction: given a short sequence of amino acids, determine its antimicrobial activity, identify the specific pathogen it targets and ascertain the minimum inhibitory concentration (MIC) needed to inhibit the pathogen. Figure 1 illustrates this concept through a hierarchical scheme of challenges:

- Level 1: Classify the input sequence as an AMP or non‐AMP. This is the most widely addressed challenge in the field. Most of the studies listed below in the shallow and deep learning sections focus on solving this challenge.
- Level 2: Classify the input sequence in a hierarchical manner or directly, distinguishing between different bioactivities. Many studies address the problem up to this level; see, for instance, the work by (Martínez‐Mauricio, García‐Jacas, and Cordoves‐Delgado 2024) and references therein.
- Level 3: A limited number of studies have reached this level, which involves higher resolution taxonomy target classification and prediction tasks, APEX (Wan, Torres, et al. 2024) is one example.
- Level 4: Only a few studies have advanced to this level, involving comprehensive analysis and prediction of AMP properties (Li, Sutherland, et al. 2022; Shao et al. 2024), in the same line, APEX (Wan, Torres, et al. 2024) predicts species specific antibacterial activity against 34 pathogens.
- Level 5: Few studies aim to reach this highest level, which involves expanding the previous level's bacteria‐specific predictions with further details, such as predicting MICs (Yan, Zhang, et al. 2023; Yao et al. 2024).

Notice that as we progress through the levels, data scarcity increases, making the challenge more difficult for Levels 3–5. In the following sections, we describe the key elements of an AI‐based approach for AMP discovery and design, highlighting the many variables yet to be explored. This discussion is preceded by a review of the relevant literature, which outlines the state‐of‐the‐art in each area.

## 3 Datasets

Numerous databases have been proposed for AMPs, though some are no longer available today. Some datasets compile and merge data from multiple sources. For instance, StarPep (Aguilera‐Mendoza et al. 2019) is a metadatabase built from a collection of 40 different databases. Currently, APD3 (Wang, Li, and Wang 2016), DBAASP V3 (Pirtskhalava, Amstrong, and Grigolava 2021) and DRAMP V3 (Shi et al. 2022) are three well‐maintained databases for bioactive peptides.

APD3 is a manually curated database consisting of 3940 peptides, 190 predicted and 314 synthetic peptides, accounting for 22 activities. DBAASP is also a manually curated and includes molecular dynamics simulations for over 3000 monomer peptides. It currently contains 22,025 peptides, of which 82% are synthetic, 14.6% are ribosomal and 3.4% are non‐ribosomal. DRAMP 3.0 contains 22,259 peptides distributed across nine activities, with 16,110 patent entries. According to an analysis by Shi et al. (2022), DRAMP 3.0 coincides with DBAASP in 3361 peptides, DRAMP 3.0 and APD share 2172 peptides, and APD and DBAASP share 1914, while the three databases share 1395 peptides.

Further analysis of the evolution of databases, from the first APD (Wang and Wang 2004) to its third version, APD3 (Wang, Li, and Wang 2016), and more recent improvements are provided by Wang (2023). Additionally, a short review of antimicrobial peptide databases and their associated computational tools is provided by Ramazi et al. (2022). We refer readers to these reviews for detailed information on the composition of the currently available databases.

### 3.1 Perspective on Databases

A significant challenge in training datasets is the selection of negative examples. One common approach is to generate random sequences with a uniform distribution. Another method is to take sequences from a protein databank like UniProt, concatenate them, and then chop them at random locations. A more biology‐driven approach (Ma et al. 2022) involves building a non‐AMP dataset by searching UniProtKB for peptides residing in the cytoplasm and removing those labelled as antimicrobial, antibiotic, antiviral, antifungal, effector or excreted. Similar approaches have been implemented by others (Torrent et al. 2011; Veltri, Kamath, and Shehu 2018; Xiao et al. 2013). However, these approaches rarely analyse the similarity between the sampled negative examples utilised in training and in benchmarking, although it is known that such similarity affects the performance of ML algorithms (Sidorczuk et al. 2022).

A crucial missing element is labelled negative sets, that is datasets of peptides known to be inactive against specific pathogens. Building such datasets would significantly enhance the robustness of predictive models. From a data science perspective, an important missing aspect in databases is an analysis of their diversity, coverage, and the statistical properties of positive and negative examples.

An important missing element in datasets associated with AMPs and antibiotics in general is the lack of experimental standardisation when building datasets, a critical requirement when creating training sets for AI models (de la Fuente‐Nunez 2024). Many parameters need to be considered for this purpose, for example the medium, pH, temperature, inoculum, strain and every experimental condition that might bias the results.

## 4 Descriptors

The first generation of AMP activity prediction relied on phenomenological approaches. In these methods, a few physicochemical descriptors were computed, and if the query peptide had these descriptors within pre‐defined bounds, it was assigned activity; otherwise, it was not. These approaches are based on understanding the underlying mechanisms that govern a phenomenon. For example, Tossi, Sandri, and Giangaspero (2000) analysed six structural and physicochemical parameters that modulate peptide activity: sequence, size, structuring, charge, amphipathicity and hydrophobicity.

The second generation of AMP activity prediction utilised physicochemical descriptors along with absolute, relative and pseudo counts of amino acid appearances in sequences to build machine learning models. There are many possible representations, some of which are based on amino acid composition and others strictly on physicochemical properties (Chen et al. 2020; Pande et al. 2023).

Despite extensive literature on feature selection, few studies address feature selection for AMP classification. This problem can be modelled as a single‐objective optimization (Beltran‐Verdugo, Del Rio‐Guerra, and Brizuela 2020) or a multi‐objective (Beltran, Aguilera‐Mendoza, and Brizuela 2018) optimization. In line with developments in natural language processing (NLP), self‐learned features emerged as a third generation of sequence representations, often tailored to specific learning models, primarily within the deep learning paradigm.

### 4.1 Perspective on Descriptors

One key question is whether self‐learned features are superior to rational physicochemical features or if they complement each other. A preliminary step in this direction was recently taken by García‐Jacas, García‐González, et al. (2022). Features derived from the protein language model ESM‐2 (Lin et al. 2023) (see section on protein language models) may soon be routinely used for various AMP‐related learning tasks. Further efforts investigating self‐learned descriptors are necessary to understand their overall comprehensiveness.

A relevant issue associated with descriptors is the lack of studies addressing the applicability domain for AMP sequence space. Pinacho‐Castellanos et al. (2021) provided one of the few examples of such analysis. Analysis of the applicability domain is routinely performed in small‐molecule research (Wang and Chen 2023). Given the discrete and irregular nature of sequence space, a profound analysis is needed to address the meaning, relevance and potential redefinition of this concept in the antimicrobial peptide field.

Another overlooked direction is the unification of chemical spaces of small molecules and peptides. Small molecules, in most cases, consist of fewer than 100 atoms and have a defined structure with low molecular weight. In contrast, peptides have an order of magnitude more atoms, with greater structural complexity and higher molecular weight. Despite these differences, both small molecules and peptides share commonalities in their ability to interact with biological targets. The first step needs to clarify to what extent both spaces can be unified. If this is possible, the benefit will be the large corpus of knowledge in the small‐molecule field that can directly supplement the peptide field.

An approach trying to exploit this idea for predicting antimicrobial activities of known human drugs is the work by Nava Lara et al. (2019). A similar work in this direction (Liu, Hopkins, et al. 2023) seeks to transform, through ML, peptide ligands for the ghrelin receptor into small‐molecule ligands. To this end, an ML model was trained with peptides and small molecules to then infer small molecules. The space unification was performed through the computation of atom‐level descriptors. With a different approach, a more recent study (Hayward and Beekman 2024) sought to convert turn‐motif and cyclic peptides into small molecules for targeting protein–protein interactions. We argue that a promising approach can be to unify the spaces at the atomic level and consider the interactions among the atoms in the molecule. However, this approach will probably be restricted to similar mechanisms of action in the analysed molecules.

## 5 Machine Learning Models

### 5.1 Shallow Learning Models

Several ML‐based methods have been used for identifying AMPs, including hidden Markov models (Fjell, Hancock, and Cherkasov 2007), random forest (Bhadra et al. 2018; Manavalan et al. 2017; Pinacho‐Castellanos et al. 2021) and support vector machines (Cao, Xu, and Liang 2013; Manavalan et al. 2017), among others. Extensive reviews of these shallow learning approaches can be found in Ramazi et al. (2022) and Yan et al. (2022). Most of these approaches address the binary classification problem across many labels, such as antibacterial, antifungal, antiviral, antiparasitic and anticancer activities. Some approaches tackle the multi‐class problem, while fewer address the multi‐label problem. Bizzotto et al. (2024) built a dataset containing peptides derived from food fermentation and compared different encoding methods and shallow learning methods to classify nine endpoints. They concluded that no single encoding and model outperforms all others over all endpoints, aligning with the well‐known no‐free‐lunch theorems (Wolpert 2002).

The computational and ML paradigm, equipped with high‐throughput experimental screening, has successfully discovered effective antimicrobial peptides in extant (Torres et al. 2022) and extinct (Maasch et al. 2023; Wan, Torres, et al. 2024) human proteomes, opening the new and exciting field of molecular de‐extinction. These studies identified the first therapeutic molecules in extinct organisms. Foundational is the random forest model trained to predict cleavage site of human proteases. Peptides resulting from this in silico digestion then undergo experimental evaluations. The potential of molecular de‐extinction, as presented by Maasch et al. (2023) and Wan, Torres, et al. (2024), is achievable due to ML approaches that should be further explored with new methods, endpoints and datasets.

A recent approach, AMPActiPred (Yao et al. 2024), uses a deep forest architecture to characterise peptide activity against diverse bacterial species, using physicochemical and relative frequency of amino acids as descriptors. The method works in three stages: predicting antibacterial activity, identifying the target species and predicting the MIC. Thus, the pipeline starts at Level 2 in our hierarchical scheme (Figure 1) and achieves Levels 4 and 5, skipping Level 3.

Another approach aimed at identifying the antibacterial bioactivity of peptides in the human gut microbiome (Torres et al. 2024) against gut commensals and pathogenic bacteria found that 70.5% of the predicted compounds had antibacterial activity.

An area that has been largely overlooked is the prediction of the mechanism of action. For instance, an ML model to detect membrane activity of AMPs was proposed by Lee, Lee, and Fulan (2017). They used an SVM classifier where peptides are represented by descriptors computed using the propy Python package (Cao, Xu, and Liang 2013). The training set consisted of 286 AMP and 286 decoy alpha‐helical peptides, with 1588 descriptors. A rational feature selection process was applied, and the 12 most predictive descriptors were kept.

### 5.2 Deep Learning Models

A recent study developed a new deep learning model, called APEX, to mine all extinct organisms known to science (the extinctome) as a source of antibiotics. This landmark study discovered thousands of new peptide molecules throughout evolutionary history and yielded preclinical candidates from the woolly mammoth, among many other organisms (Wan et al. 2023; Wan, Torres, et al. 2024). APEX was trained on an in‐house dataset and DBAASP. The pipeline utilises an encoder neural network, combining recurrent and attention neural networks for feature extraction from peptide sequences. The encoder neural network was then coupled with multiple downstream neural networks to predict antimicrobial activity. The pipeline identified 37,176 molecules with predicted antimicrobial activity. Of these, 11,035 were classified as archaic compounds and not currently found in extant organisms. Many of the compounds were effective both in vitro and in two different preclinical mouse models with activity of the lead hits being comparable to the last‐resort antibiotic polymyxin B. Molecules discovered by APEX, such as mammuthusin, mylodonin, elephasin, megalocerin and hydrodamin, are prime examples of preclinical antibiotic candidates discovered using DL.

DL methods have also been used to mine the tree of life as a source of antibiotics, including in microbial dark matter (bacterial species that are not easily cultured in a laboratory setting) through exploration of the global microbiome (Santos‐Júnior et al. 2024). In this work, the team computationally mined the global microbiome (63,410 metagenomes and 87,920 microbial genomes) and discovered nearly 1 million new antibiotic molecules, several of which were effective in a preclinical mouse model. This is the largest exploration described of biological data as a source of antibiotics. A peptide was approved for synthesis if, in addition to fulfilling six criteria, it was also predicted to have AMP activity with a suite of six DL methods, including AMPScanner (Veltri, Kamath, and Shehu 2018), AMPLify (Li, Sutherland, et al. 2022), as well as four others.

A review by Yan et al. (2022) presents an exhaustive description of DL approaches for AMP classification. While it also covers SL methods, a quantitative comparison between the two paradigms is missing. This gap is addressed by García‐Jacas, Pinacho‐Castellanos, et al. (2022) who compare both deep and shallow learning approaches. For example, using the same dataset used to train and test AMPScanner (Veltri, Kamath, and Shehu 2018), a simple shallow learning model combining LogitBoost and random forest (García‐Jacas, Pinacho‐Castellanos, et al. 2022) with 67 features achieved a Matthews Correlation Coefficient (MCC) of 0.913, outperforming the deep learning‐based AMPScanner, which achieved an MCC of 0.820. Similarly, in the case of datasets used to develop Deep‐AmPEP30 (Yan et al. 2020), a LogitBoost + RF model with 145 features achieved an MCC of 0.766, compared to the 0.543 achieved by the deep learning model Deep‐AmPEP30. However, there are instances where DL models outperform SL models. For instance, AniAMPpred (Sharma et al. 2021), a deep learning model, achieved an MCC of 0.937, slightly higher than the 0.935 achieved by a LogitBoost + RF model with 81 features. Overall, a Bayesian analysis of results from both DL and SL models on the same datasets revealed a probability of 94.9% that SL models outperform DL models, compared to a 4.6% probability of DL models outperforming SL models. These comparisons underscore that, given the current amount of available data, SL models often exhibit superior performance in AMP prediction tasks. Their study concludes that, with the currently available datasets, SL approaches are preferable due to their simplicity and potential for explainability. However, the dataset size required to prefer DL over SL remains unknown. Deep learning encompasses a broad class of models that vary in representation and architecture. The following is a non‐exhaustive list of DL models applied to AMPs.

#### 5.2.1 Graph Neural Networks

Graph neural networks (Scarselli et al. 2009) have emerged as powerful learning tools to capture complex interrelations among components in various subjects of study. For an extensive survey on GNNs, see Han et al. (2024) and Wu et al. (2021). While GNNs have been widely applied to many learning tasks in protein bioinformatics (Zhang et al. 2021) in general, and in protein–protein interactions (Zeng et al. 2024) in particular, their application to AMP discovery and design is still in its early stages (Fernandes et al. 2023).

**FIGURE 2:**
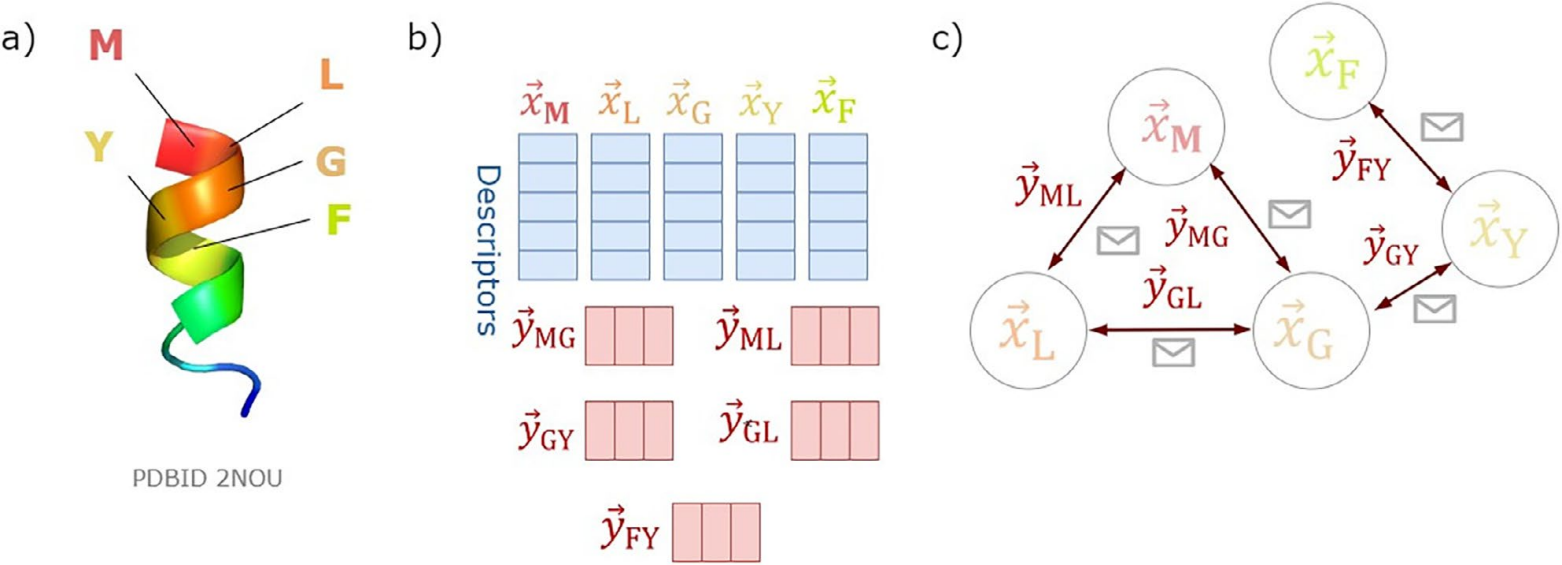
Graph neural network for AMPS. (a) Input peptide MLGYF, PDB Id 2NOU. (b) Node ($\vec{x}$) and edge ($\vec{y}$) descriptors of variable size. (c) A graph representation for the peptide MLGYF.

As shown in Figure 2, the input peptide can be represented by its amino acid sequence and derivations made from it, including its 3D structure. LABAMPsGCN (Sun et al. 2022) is a graph convolutional network (GCN) where each sequence is decomposed into *k*‐mers to build the graph. There is a node for each *k*‐mer and a node for the peptide. An edge connects two *k*‐mers if they are within a threshold distance in the peptide. Additionally, there is an edge between the node representing the peptide and every node representing the *k*‐mers derived from it. The model was trained to discriminate sequences with anti‐lactic‐acid bacteria activity from those without, addressing the Level 4 challenge.

AMPs‐Net (Puentes et al. 2022) is a GCN that encodes each peptide as a graph. In this graph, a node represents an atom (a heavy atom) and the edges represent the bonds between them. This representation captures topological information rather than the 3D structure of the peptide. Each node's feature vector consists of nine elements, including properties like charge, number of hydrogens and aromaticity. The edges also have a feature vector of three elements: bond type, conjugation and stereochemistry. This model is applied to discriminate AMPs from non‐AMPs, and to identify antimicrobial activities such as antibacterial, antifungal, antiviral and antiparasitic, addressing Level 1 and Level 2 challenges.

The first application of GNNs to discriminate AMPs from non‐AMPs using predicted structural information is sAMPred‐GAT (Yan, Lv, et al. 2023). This model uses a graph attention network (GAT) that leverages TrRosetta to predict the 3D structure of peptides. In this framework, each residue is represented by a node in the graph and two nodes are connected if the distance between their beta carbons is equal to or less than 20 Å. Each node features a vector composed of information associated with its corresponding amino acid.

Inspired by the powerful representations of ESM‐2 models and previous work, a recent study by Cordoves‐Delgado and García‐Jacas (2024) merged these approaches to propose esm‐AxP‐GDL, achieving outstanding results with an AUC value over the test set of 0.99 on the binary classification task of AMP versus non‐AMP. The authors trained a GAT on a dataset of 67,058 peptides, with their 3D structure predicted by ESMFold (Lin et al. 2023). In this model, each amino acid is represented by a node, and two nodes are connected by an edge if their corresponding alpha carbons are within a specified threshold distance. Each node has a feature vector of size 1280, derived from a 33‐layer model of ESM‐2 (Lin et al. 2023).

GNNs can capture topological and geometrical representations of peptides, as well as more complex relationships at the sequence level. However, the optimal representation model remains unclear: it is not known whether each node should represent an atom, an amino acid or a *k*‐mer. Similarly, questions arise regarding the edges. Even with a fixed model, it is uncertain what the best feature vectors for nodes and edges are and how this selection affects the message‐passing model used. Further research is needed to optimise these choices and improve model performance.

#### 5.2.2 Protein Language Models

Under the DL paradigm, a new trend in NLP known as large language models (LLM) has emerged. LLMs are scaled‐up versions, both in model size and data size, of pre‐trained language models designed to model and solve complex learning tasks by generating text (Zhao et al. 2023). These models are built using deep learning architectures, particularly transformers (Vaswani et al. 2017). Examples of LLMs include OpenAI's GPT (Generative Pre‐trained Transformer) series, including GPT‐4, Google's BERT (Bidirectional Encoder Representations from Transformers), XLNet and T5 (Text‐to‐Text Transfer Transformer).

**FIGURE 3:**
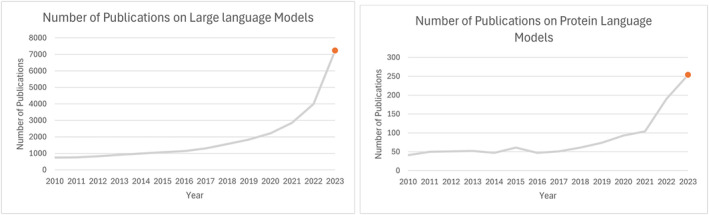
(a) Number of publications per year of works on LLMs. Query = ‘Large Language Models’. (b) Number of publications per year of works on Protein LMs. Query = ‘Protein Language Models’.

Although LLMs have been successful in NLP (see Figure 3a), their application to the protein world is still in its infancy (see Figure 3b), yet they are already showing promising results. The data in the graph, obtained from the Scopus database, indicate that there are still many approaches in NLP that are yet to be explored in the protein field in general and in the area of AMPs in particular.

Large protein language models extract valuable information hidden in the sequences of all known proteins (Bepler and Berger 2021). Several pre‐trained deep neural language models have been developed for protein sequences, including ESM (Rives et al. 2020), ProteinBERT (Brandes et al. 2022), ProtTrans (Elnaggar et al. 2022) and ProtGpt2 (Ferruz, Schmidt, and Höcker 2022).

The first protein transformer language model, ESM‐1 (Rives et al. 2020) was soon improved by ESM‐1b, increasing the number of layers from 12 to 33 and performing an extensive hyperparameter sweep. ESM‐1b was pre‐trained on UniRef50 and contains 650 million parameters. The following version, ESM‐2 (Lin et al. 2023), was trained on the UR50/D2021_04 dataset. ESM models revolutionised the field by simplifying the complex, multi‐stage process of predicting protein contact maps to a single forward pass of a pre‐trained protein language model, significantly improving state‐of‐the‐art methods for this task. The last version of ESM, ESM‐3 (Hayes et al. 2024) is a multimodal generative language model that includes information from sequences, structures and function. The model was capable of generating new fluorescent proteins.

ProteinBERT (Brandes et al. 2022) was pre‐trained on approximately 106 million sequences from UniProtKB/UniRef90, encompassing the entire tree of life. Each protein is associated with its amino acid sequence and GO annotation from UniProtKB. The model included 8943 GO annotations that appeared at least 100 times in UniRef90. For protein representation learning, the self‐supervised part of the model was trained with corrupted amino acids in the sequence and corrupted GO terms, aiming to recover their uncorrupted versions. ProteinBERT was tested on nine endpoints, including protein function, structure, post‐translational modifications and biophysical properties.

ProtTrans (Elnaggar et al. 2022) trained two autoregressive models and four auto‐encoder models by leveraging sequences from UniRef. ProtTrans is intended to exploit the main feature of each of these six models. The pre‐trained embeddings from unlabeled data captured the biophysical properties of the sequences. These transformer‐based approaches produced competitive results on various supervised learning tasks, such as predicting subcellular location, water solubility and secondary structure.

ProtGpt2 (Ferruz, Schmidt, and Höcker 2022) is an autoregressive transformer model with 36 layers and 738 million parameters that efficiently generates protein sequences. It was trained on 50 million protein sequences from UniRef50.

#### 5.2.3 Large Language Models for AMPs

LLMs for AMPs have been used to build both discriminative and generative models, see the Table 1 below for an overview. These models leverage the capabilities of LLMs to address complex tasks in AMP research, including the classification and generation of novel AMP sequences.

**TABLE 1:** Overview of discriminative and generative models in AMP research.

Method	Description	Application	References
*Discriminative models*			
LMPred	It is built on BERT, XLNet and T5 with a CNN for fine‐tuning	Classification AMP vs. non‐AMP	Dee (2022)
UniDL4Biopep	Uses ESM‐2 for embedding and CNN for fine‐tuning	Classification performance on 20 endpoints	Du et al. (2023)
PeptideBERT	Uses ProteinBERT for embedding and MLP for fine‐tuning	Classification performance on 3 endpoints	Guntuboina et al. (2023)
AutoPeptideML	Uses ESM for embedding and SVM, RF, for fine‐tuning	Classification performance as a function of embedding size on 18 endpoints	Fernandez‐Diaz et al. (2024)
FusPB‐ESM2	Combines ProBERT and ESM‐2 for embedding and a simple NN for fine‐tuning	Classification of CPP vs. non‐CPP	Zhang, Li, et al. (2024)
Pang's approach	Uses pre‐trained BERT for embedding and MLP for fine‐tuning	Multi‐label classification for 7 endpoints	Pang et al. (2022)
*Generative models*			
FBGAN	GAN + ESM‐2, uses feedback in the generative process	Generates 5000 peptides and compares performance with AMPGAN and HydrAMP	Zervou et al. (2024)
RNN + LLM	LSTM + ProtTrans embedding trained on DBAASP and fine‐tuned on anti‐*C. acnes*	42 novel AMPs against *C. acnes* with MIC of 2–4 μg/mL	Dong et al. (2024)
AMP‐diffusion	ESM‐2 to generate the embedding	The generated sequences resemble real AMPs in terms of perplexity, diversity and PC properties	Chen et al. (2023)
ProT‐Diff	The diffusion model is placed between the encoder and decoder of ProtT5	The authors filtered by MIC 35 peptides, out of thousands, 34 of them showed antibacterial activity	(Wang et al. 2024)

##### 5.2.3.1 Discriminative Models

**FIGURE 4:**
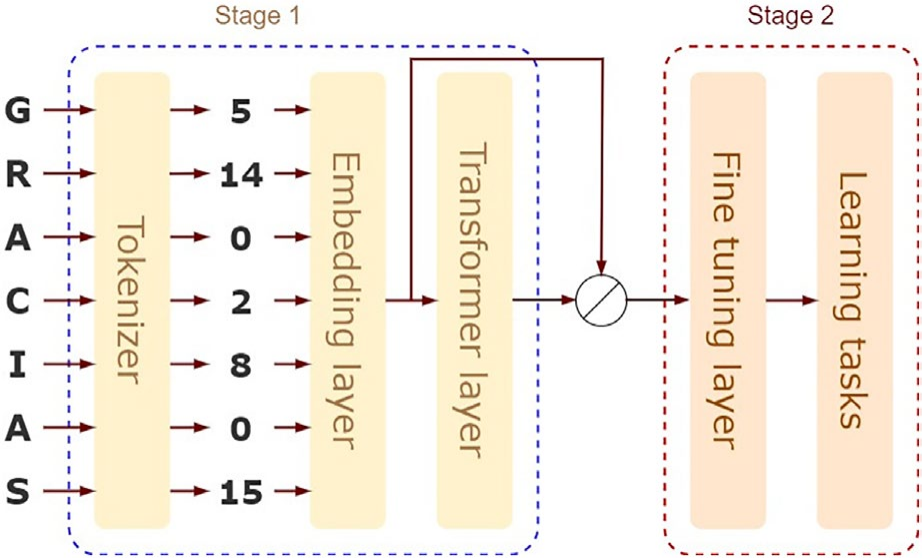
General framework for the use of LLMs in the peptide context. Stage 1 consists of a tokenizer, embedding layer and the transformer layer (this layer is absent in some approaches). Stage 2 receives the output of the embedding layer or of the transformer layer, then by using this representation, the fine‐tuning layer is trained with for the specific learning task such as antimicrobial activity discrimination.

Building on the success of general LLMs in NLP and their application to proteins, LMPred (Dee 2022) emerged as one of the first LLMs for peptide activity prediction. It uses a pre‐trained model based on a large protein space context. LMPred is built on BERT (Devlin et al. 2018), XLNet (Yang et al. 2019) and T5 (Raffel et al. 2020) models pre‐trained on the UniRef100 and UniRef50 datasets to generate contextualised embedding in protein space. A standard CNN architecture is used downstream for fine‐tuning antimicrobial activity classification (AMP vs. non‐AMP) on two datasets (see the fine‐tuning layer in Figure 4).

UniDL4Biopep (Du et al. 2023) is a universal DL architecture for transfer learning in binary classification tasks for peptides. It includes a pre‐trained protein language model to predict peptide bioactivities, utilising the ESM‐2 model with six layers and 8 million parameters for a 320‐dimensional embedding. This embedding is coupled with a CNN architecture for specific learning tasks (see Figure 4 for the fine‐tuning layer). The model was evaluated on 20 binary classification endpoints, including antimicrobial activities, neuropeptide activity, toxicity, anticancer activity and others. Previous work by (García‐Jacas, García‐González, et al. 2022) used the ESM‐1b embedding (1280‐ dimensional) for similar tasks, focusing on antimicrobial activity and coupling the embeddings with classical ML approaches like Random forest for fine‐tuning (Figure 3). Both studies provide evidence of the relevance and completeness of the information contained in the embeddings.

PeptideBERT (Guntuboina et al. 2023) is a protein language model based on ProteinBERT and fine‐tuned for tasks such as predicting haemolysis, solubility and non‐fouling properties. Built on ProBERT, a transformer with 12 attention heads and 12 hidden layers, PeptideBERT leverages over 106 million sequences from UniProtKB/UniRef90 during its pre‐training phase. An MLP layer is added for fine‐tuning the three endpoints: solubility, haemolysis and non‐fouling.

AutoPeptideML (Fernandez‐Diaz et al. 2024) offers an automated machine learning solution for developing predictors of peptide bioactivity. It uses embedding generated by ESM for peptide representation (Stage 1, Figure 4) and employs classical ML models like support vector machine, *k*‐nearest neighbours, random forest, among others, for fine‐tuning (Stage 2, Figure 4). The vector representation for each amino acid is of size 320, which is also the representation for the peptide averaged over all its amino acids. The model overall has 8 million parameters. Innovations include the generation of negative samples and an analysis of the impact of embedding size on model performance, with a total of 18 endpoints such as antibacterial, anticancer, antifungal, antimalarial and blood–brain barrier penetrator. The authors found that increasing the embedding size did not significantly impact algorithm performance, justifying the use of smaller embeddings.

FusPB‐ESM2 (Zhang, Li, et al. 2024) combines ProBERT and ESM‐2 pre‐trained models to extract and merge 1024 features for each peptide (Stage 1, Figure 4). The ESM‐2 model produced 480 features, then with a two‐layer NN, a vector of 1024 features was generated. The merged features are then used to train a neural network with 1024 inputs and two outputs (cell‐penetrating peptides, CPP and non‐cell‐penetrating peptide, non‐CPP) (Stage 2, Figure 4).

Pang and colleagues addressed seven classes and multi‐label scenarios using a transfer learning approach (Pang et al. 2022). They employed a pre‐trained BERT model from the TAPE archive (Rao et al. 2019) for representation generating (Stage 1, Figure 4), utilising the Pfam dataset (Mistry et al. 2021) with 31 million sequences. An MLP was used for fine‐tuning (Stage 2, Figure 4), handling both the binary classification to discriminate AMPs from non‐AMPs and multi‐label classification tasks, with varying numbers of layers. This work is one of the few addressing multi‐label classification, that is, the task of predicting more than one activity in a single inference step. The simultaneous prediction targets are anti Gram‐negative, anti Gram‐positive, antifungal, antiviral, anticancer, antiparasitic and mammalian inhibition.

##### 5.2.3.2 Generative Models

An overview on deep generative models for peptide design was recently presented by Wan, Kontogiorgos‐Heintz, and de la Fuente‐Nunez (2022). The paper categorises generative models into neural language models (NLM), variational autoencoders (VAEs) and generative adversarial networks (GANs).

An example of using a transformer, transfer learning and standard classification is demonstrated in the work by (Dong et al. 2024). They used a simple generative model to design new antimicrobial peptides against *Cutibacterium acnes*. To increase the number of training sequences, a phylogenetic tree was built to retrieve closely related bacteria species to *C. acnes*, with DBAASP (Pirtskhalava, Amstrong, and Grigolava 2021) as the main source for recovering the training data. A recurrent neural network (RNN) was used as a generator, trained on non‐specific peptides from DBAASP and fine‐tuned with recovered anti‐*C. acnes* and related species. The classification stage was performed with various models such as gated recurrent unit (GRU; Bahdanau, Cho, and Bengio 2014), long short‐term memory (LSTM; Hochreiter and Schmidhuber 1997) and classifiers based on ESM (Lin et al. 2023) and ProtTrans (Elnaggar et al. 2022) embeddings. The authors found that the best results were achieved with a combination of LSTM and ProtTrans. A set of 42 novel linear peptides was generated and then synthesised for experimental evaluation. Five of them showed high potency and selectivity against *C. acnes* with a minimum inhibitory concentration of 2–4 μg/mL.

Recently (Zervou et al. 2024) included feedback in the generative process with their FBGAN method. This approach uses a GAN and incorporates a classifier during training, which can be either on *k*‐mers or utilise transfer learning from the ESM‐2 model. The performance of FBGAN was compared with AMPGAN (Oort et al. 2021) and HydrAMP (Szymczak et al. 2023). In the task of generating 5000 sequences, both versions of FBGAN outperformed HydrAMP and showed comparable performance to AMPGAN. Performance was measured using the edit distance to real sequences and sequence identity to the real dataset.

A powerful generative paradigm known as diffusion or score‐based generative models has recently shown outstanding performance in computer vision applications (Ho, Jain, and Abbeel 2020). Originally designed for continuous data, these models have been extended to discrete data, including NLP (Li, Thickstun, et al. 2022). They have already shown notable results in computational biology (Guo et al. 2023). For instance, in protein design, promising results have been achieved with RFDiffusion, which was used to design symmetric assemblies, metalloproteins and protein binders (Watson et al. 2023). The basic idea of the diffusion model for sequences consists of converting the discrete nature of sequence space to a continuous space using an embedding. Then, the standard forward process gradually adds noise to the target variable, and the reverse process (a denoising step) recovers the target variable. A rounding step converts the continuous recovered variable into discrete text, performed by taking the most probable text at each position (Li, Thickstun, et al. 2022), such as a word in NLP or an amino acid in the protein language.

In the peptide domain, the application of diffusion models is still in its infancy. The first proposal, AMP‐diffusion (Chen et al. 2023), uses ESM‐2 to generate a continuous embedding from the input sequence and applies the diffusion process over this continuous latent space. The process then denoises this latent vector representation, converting it back to a new sequence. The authors showed that the generated sequences resemble experimentally validated AMPs in terms of perplexity, diversity and physicochemical properties.

Another recent proposal, ProT‐Diff (Wang et al. 2024), combines a diffusion model with a protein language model into a single neural network for generating novel AMPs. In this work, a diffusion model called ProT‐Diff is placed between the encoder and decoder components of a transformer‐based language model called ProtT5‐XL‐UniRef50. In this configuration, the diffusion model can be trained solely in the latent‐space representation of peptide sequences using a dataset of AMPs with the desired characteristics, then the ProT5 decoder converts the discrete sequences to a continuous embedding. In the generation process, the diffusion model can recover a valid latent‐space representation from input Gaussian noise, and then utilise the ProtT5 decoder module to reconstruct the peptide sequence from the recovered continuous vector. The method generated thousands of AMPs; the authors selected 35 of them for synthesis. Of these selected peptides, 34 showed antibacterial activity against Gram‐positive and Gram‐negative bacteria, and in vivo activity against a clinically relevant drug‐resistant *E. coli* strain. The selection of the 35 peptides was done by filtering those with a predicted MIC of less than 10 μM.

In Table 1, a brief overview of the discriminative and generative models is presented.

### 5.3 Perspective on ML Models

The machine learning techniques described thus far are generating an abundance of peptides with antimicrobial activity. However, a peptide's ability to inhibit bacterial growth is only one aspect of a clinically applicable antibiotic. Other important pharmacological properties, such as toxicity, solubility, bioavailability and often the peptide's mechanism of action, must be thoroughly researched when translating hits into medicine. The experiments necessary to elucidate these properties are far more intensive than initial hit discovery experiments, often requiring additional months or years of dedicated research. Therefore, recent machine learning tools have been developed to predict these properties, helping researchers prioritise hit candidates for follow‐up experiments.

There is currently a lack of literature on predictions of mechanisms of action, likely due to the narrow range of mechanisms shown in currently studied AMPs. However, as machine learning methods continue to broaden our knowledge of novel peptides with antibacterial properties, the utility of mechanism of action predictors will only continue to grow. A comprehensive review of the techniques used to elucidate the mechanism of action for AMPs, mainly by interaction with the bacterial membrane, was recently presented by Schäfer and Wenzel (2020). The experiments outlined are resource‐intensive and challenging to conduct in meaningful high‐throughput. For instance, among spectroscopic approaches, solid‐state NMR is the technique of choice to analyse the location of the peptide within the membrane (Fillion and Auger 2015), but NMR machines are not widely accessible. This limitation simultaneously motivates the generation of more data related to explaining the mechanisms of action of AMPs.

Toxicity prediction of AMPs (Wei et al. 2022) is another urgent need to bring the AMPs closer to the clinic. One approach used DBAASP to train a generative model for producing AMPs (Capecchi et al. 2021). In this work, a classifier was then used to test for activity as well as toxicity. They generated 28 peptides, each at least five mutations away from peptides in the database. Of these, 12 exhibited antimicrobial activity, and 8 were also non‐haemolytic, demonstrating an encouraging effectiveness rate of over one third.

Explainability is another overlooked aspect for AMPs discovery; there is still scarce literature on this relevant subject that might help pave the way for understanding these peptides. A recent example (Bhatnagar et al. 2024) predicts antibacterial activity, haemotoxicity and efficacy against *Staphylococcus aureus* , *Escherichia coli* and *Pseudomonas aeruginosa* for any query peptide. The predictor is based on physicochemical descriptors along with sequence composition to train a support vector machine for binary classification. The approach applies SHAP analysis for explainability to determine the most relevant physicochemical properties and amino acid composition to explain the antibacterial activity, efficacy and haemolytic activity.

## 6 Optimisation of ML‐Based Approaches

**FIGURE 5:**
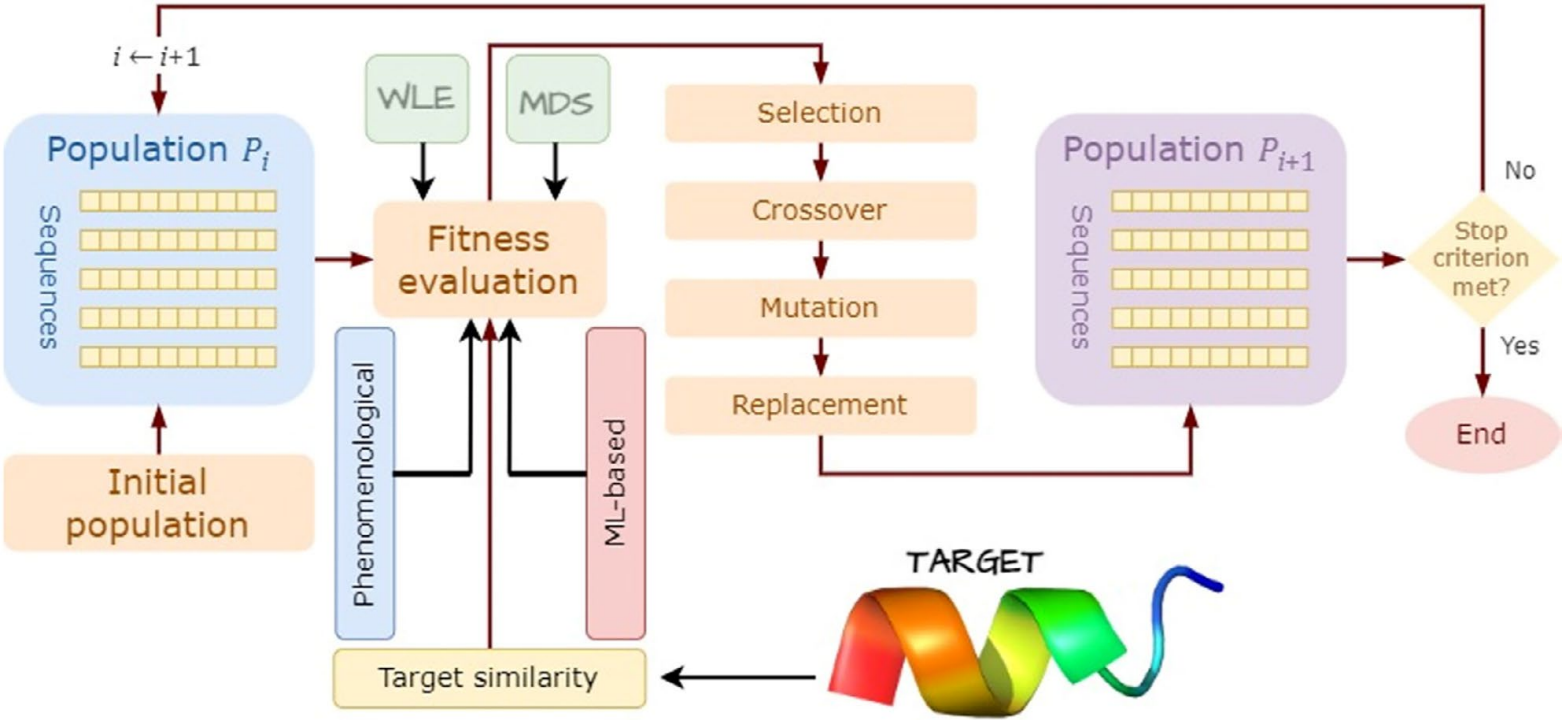
General scheme for evolutionary optimisation‐based approaches. The fitness function can be related to physicochemical properties the design wants to achieve (phenomenological). The fitness can be guided by an ML repressor (ML‐based), or it can use some target information (Target similarity). WLE stands for Wet Lab Experiments and MDS for molecular dynamics simulation. The fitness function can also utilise any combination of the three approaches.

To explore the vast sequence space of peptides, evolutionary algorithms guided by different fitness functions have been introduced. The fitness functions are key to these approaches. One of the first methods, under the ML‐based fitness computation in Figure 5, is based on an evolutionary multi‐objective optimisation algorithm (Maccari et al. 2013). The approach uses QSAR‐based features to train a random forest algorithm and with objectives and constraints according to the design target. As shown in Figure 5, fitness can also be calculated using a phenomenological approach. For instance, under a multi‐objective paradigm, the distances with respect to a centroid of a hypercube of four physicochemical properties, taken from del Rio et al. (2001), were used as optimisation criteria by Beltran and Brizuela (2016).

A landmark study by Porto et al. (2018) utilised an evolutionary algorithm to generate synthetic peptides with demonstrated anti‐infective efficacy in preclinical mouse models. Fragments of guavanins were used as the initial population. This research highlighted the potential of computational approaches in creating effective antibiotics for realistic models, paving the way for further advancements in using computer algorithms for antibiotic discovery. Notably, the algorithm used in this study produced molecules that targeted bacteria through a novel mechanism, membrane hyperpolarisation, which is distinct from the depolarising action of most peptide antibiotics. This experiment exemplifies the emergent properties of computational approaches in biological systems. Fragments of guavanins were used as initial population.

Another novel approach included wet lab experiments (WLE in Figure 5), into the fitness evaluation loop (Yoshida et al. 2018). With just three rounds of the evolutionary process, a 160‐fold increase in potency was achieved. The antimicrobial activities of the peptides were evaluated in vitro by measuring the half‐maximal inhibitory concentration (IC_50_) against an *E. coli* strain. This in vitro data were then used to train a linear regression model to analyse amino acid substitutions and their corresponding changes in IC_50_. The computed and estimated values were included in a fitness matrix.

In a recent multi‐objective optimisation approach (Liu, Zhang, et al. 2023), peptide activity and population diversity were optimised simultaneously. The peptide activity was predicted using a DL model (ML‐based in Figure 5). The generated AMPs were predicted to have high potency and were distinct from each other and from AMPs in the used datasets.

An almost unexplored approach for peptide design in this optimisation context is to use the 3D backbone of a target peptide (see Figure 5) as input, generate a sequence, use a structure predictor algorithm and compare the predicted structure with the target backbone. The differences are then fed back into the sequence generator. A closely related method, following an optimisation approach, was recently proposed by Moffat, Greener, and Jones (2021). In this method, the input is the 3D coordinates of the backbone atoms, and the output is a sequence of amino acids that, upon folding, minimise the *L*_dist_, loss function used in AF2. The proposed approach uses a generative neural network to produce 1000 sequences, which are then ranked based on their TM‐Score. The top‐ranked sequence is subjected to a single amino acid mutation process, where after each mutation, the structure of the mutant is predicted using AF2. This iterative process is guided by the *L*_dist_ score and continues for up to 20,000 iterations. This method exemplifies how structure‐guided design can be practically implemented to generate novel peptides with desired structural properties. Similar ideas should be explored for the design of both structured and unstructured peptides. This approach could potentially compete with diffusion models, as the desired properties of the design can be included in the objective function and measured at every step of the optimisation process.

## 7 Summary of Positive Impacts of the Analysed Methods in the Discovery of New AMPs

### 7.1 Speed and Efficiency in AMP Screening

The rapid in silico screening of large peptide libraries has significantly accelerated the discovery process. For instance, both extant (Torres et al. 2022) and extinct (Maasch et al. 2023; Wan, Torres, et al. 2024) proteomes can now be computationally screened within hours. Similarly, the human gut microbiome has been efficiently screened (Torres et al. 2024), paving the way to explore a broad spectrum of species in a reasonable timeframe.

### 7.2 Cost‐Effectiveness

The combination of rapid screening and improved classification accuracy directly contributes to cost‐effective discovery of new AMP hits. This efficiency reduces the resources required for experimental validation and accelerates the overall development pipeline.

### 7.3 Generative Capabilities

Advances in generative models now enable the design of novel AMPs at a low cost, allowing the exploration of previously unknown chemical spaces. This opens opportunities for creating innovative AMP candidates with potentially enhanced therapeutic properties.

## 8 Perspective on Urgently Needed Experiments

Currently, works predicting minimum inhibitory concentrations (MICs) for AMPs (Level 5 in Figure 1) are limited. To significantly impact the development of in silico prediction methods, it is imperative to conduct experiments that determine MICs for specific bacterial species. This experimental data are crucial for validating new predictive models and ensuring that the computationally predicted AMPs are effective in vitro. Furthermore, MIC assays tailored to directed single amino acid mutations can provide high‐resolution data, enhancing the development of more precise predictors. By starting with a known AMP and determining its MIC, as well as the MICs for its mutants, we can gain deeper insights into structure–activity relationships. Similar assays for toxicity measurements are also essential. Collecting data from these experiments is urgently needed to pave the way for more meaningful and accurate predictions in AMP discovery.

## 9 Conclusions and Future Prospects

One important drawback of AMPs is their poor in vivo toxicity performance (Chen and Lu 2020; Greco et al. 2020; Lim et al. 2010). This issue may arise from the fact that only a single endpoint is often considered in the classification process. Incorporating additional endpoints, such as the immunomodulatory activities of the peptides, probability of proteolytic degradation and likelihood of binding to serum proteins, could be beneficial. For instance, some peptides exhibit in vitro activity against bacteria but show no in vivo activity against the same bacteria (Tuxpan‐Pérez et al. 2022). Developing easy experimental proxies to validate favourable immunomodulatory effects will significantly impact the discovery of new therapeutic AMPs. Studies identifying which immunomodulatory properties synergise with the antimicrobial activity of peptides and which properties counteract it are still needed and could be crucial for designing successful AMPs.

Incorporating biological knowledge is essential to improving protein language models (Bepler and Berger 2021). Recent work (Ding and Steinhardt 2024) shows that pLMs are biased by unbalance sequence sampling across species. Additionally, Aguilera‐Puga et al. (2024) highlight the experimental bias introduced in biological experiments. For example, in vitro assays are typically performed with bacterial strains that are easy to grow and obtain, but studies involving clinical isolates are lacking, making computational methods agnostic to these situations.

As the work of Veltri, Kamath, and Shehu (2018) on deep learning (DL) for AMP classification, many variants have emerged (Hamid and Friedberg 2019). However, it has been demonstrated that for a set of DL model built on a given dataset, a shallow learning (SL) model can be constructed to generate comparable or better performance than the DL counterpart (García‐Jacas, Pinacho‐Castellanos, et al. 2022). This suggests that the number of available peptides is not yet sufficient to fully exploit the generalisation power of DL models. Fair comparisons between shallow and deep models, considering the training and testing datasets used, are needed to support this non‐mainstream claim.

With the advent of new and accurate protein structure predictors like AlphaFold 2 (Jumper et al. 2021), ESMFold (Lin et al. 2023), HighFold (Zhang, Zhang, et al. 2024), PepFold (Maupetit, Derreumaux, and Tuffery 2009), RoseTTAFold (Baek et al. 2021) and OmegaFold (Wu et al. 2022), a new paradigm of structure‐guided design of AMP is possible. This approach is particularly clear when designing structured peptides but may also benefit linear peptides. Structure‐guided design is worth exploring even in these cases. For an extensive review of peptide structure prediction methods and tools, see Wu et al. (2024).

The field of AMP discovery and design would benefit from a community‐wide competition similar to CASP (Critical Assessment of protein Structure Prediction) or CAFA (Critical Assessment of Function Annotation) for predicting and designing peptide activities. One scenario could involve predicting whether a given peptide will kill or inhibit a pathogen and providing an interval for its minimum inhibitory concentration (MIC). Alternatively, competitors could be given a pathogen as input data and asked to provide *k* candidate peptides predicted to kill the pathogen. The disadvantage of such a competition is that it would need to be conducted in real‐time, as the experiments are usually not overly complicated. Once a peptide's specific bioactivity is known, predicting its mechanism of action, toxicity and immunomodulatory effects would be highly useful.

## Author Contributions

**Carlos A. Brizuela:** conceptualization, investigation, writing – original draft, writing – review and editing. **Gary Liu:** writing – original draft, writing – review and editing. **Jonathan M. Stokes:** writing – review and editing. **Cesar de la Fuente‐Nunez:** conceptualization, investigation, writing – original draft, writing – review and editing.

## Conflicts of Interest

Cesar de la Fuente‐Nunez provides consulting services to Invaio Sciences and is a member of the Scientific Advisory Boards of Nowture S.L., Peptidus and Phare Bio. De la Fuente is also on the Advisory Board of the Peptide Drug Hunting Consortium (PDHC). The de la Fuente Lab has received research funding or in‐kind donations from United Therapeutics, Strata Manufacturing PJSC and Procter & Gamble, none of which were used in support of this work. Other authors have no conflicts to declare. Jonathan Stokes is co‐founder and CSO of Stoked Bio.

## Data Availability

The authors have nothing to report.
